# Using Rapid Prototyping to Develop a Cell-Based Platform with Electrical Impedance Sensor Membranes for In Vitro RPMI2650 Nasal Nanotoxicology Monitoring

**DOI:** 10.3390/bios14020107

**Published:** 2024-02-18

**Authors:** Mateo Gabriel Vasconez Martinez, Eva I. Reihs, Helene M. Stuetz, Astrid Hafner, Konstanze Brandauer, Florian Selinger, Patrick Schuller, Neus Bastus, Victor Puntes, Johannes Frank, Wolfgang Tomischko, Martin Frauenlob, Peter Ertl, Christian Resch, Gerald Bauer, Guenter Povoden, Mario Rothbauer

**Affiliations:** 1 Institute of Applied Synthetic Chemistry, Faculty of Technical Chemistry, Technische Universitaet Wien, Getreidemarkt 9/163, 1060 Vienna, Austria; mateo.martinez@tuwien.ac.at (M.G.V.M.); eva.reihs@tuwien.ac.at (E.I.R.); konstanze.brandauer@tuwien.ac.at (K.B.); patrick.schuller@tuwien.ac.at (P.S.); martin.frauenlob@tuwien.ac.at (M.F.); peter.ertl@tuwien.ac.at (P.E.); 2 Karl Chiari Lab for Orthopaedic Biology, Department of Orthopedics and Trauma Surgery, Medical University of Vienna, Währinger Gürtel 18-22, 1090 Vienna, Austria; 3 Catalan Institute of Nanotechnology, UAB Campus, 08193 Barcelona, Spain; neus.bastus@icn2.cat (N.B.); victor.puntes@icn2.cat (V.P.); 4 Institute of Chemical Technologies and Analytics, Faculty of Technical Chemistry, Vienna University of Technology, Getreidemarkt 9/164, 1060 Vienna, Austria; johannes.frank@tuwien.ac.at (J.F.); wolfgang.tomischko@tuwien.ac.at (W.T.); 5 Science, Research, and Development Division, Austrian Federal Ministry of Defence, 1090 Vienna, Austria; 6 CBRN-Defence-Centre, Austrian Armed Forces, 2100 Korneuburg, Austria; 7 Institute of Inorganic Chemistry, University of Technology, Stremayrgasse 9/IV, 8010 Graz, Austria; 8 Department for Legal Philosophy, Law of Religion and Culture, University Vienna, Freyung 6, 1010 Vienna, Austria; 9 Institute of Environmental Biotechnology, University of Natural Resources and Life Sciences (BOKU), IFA Building 1, Konrad-Lorenz-Straße 20, 3430 Tulln an der Donau, Austria

**Keywords:** environmental monitoring, nasal epithelial cells, nanotoxicology, lab-on-a-chip, impedance sensors, zinc oxide toxicity

## Abstract

Due to advances in additive manufacturing and prototyping, affordable and rapid microfluidic sensor-integrated assays can be fabricated using additive manufacturing, xurography and electrode shadow masking to create versatile platform technologies aimed toward qualitative assessment of acute cytotoxic or cytolytic events using stand-alone biochip platforms in the context of environmental risk assessment. In the current study, we established a nasal mucosa biosensing platform using RPMI2650 mucosa cells inside a membrane-integrated impedance-sensing biochip using exclusively rapid prototyping technologies. In a final proof-of-concept, we applied this biosensing platform to create human cell models of nasal mucosa for monitoring the acute cytotoxic effect of zinc oxide reference nanoparticles. Our data generated with the biochip platform successfully monitored the acute toxicity and cytolytic activity of 6 mM zinc oxide nanoparticles, which was non-invasively monitored as a negative impedance slope on nasal epithelial models, demonstrating the feasibility of rapid prototyping technologies such as additive manufacturing and xurography for cell-based platform development.

## 1. Introduction

Nanoparticles (NPs) are increasingly found in consumer products and the environment, raising concerns about their potential health impacts. As of 2021, over five thousand products contained nanoparticles. Nanoplastics are prevalent in various ecosystems, impacting marine species and potentially entering the human food chain [1,2]. Environmental nanoparticles can affect aquatic systems, causing behavioral changes and immune reactions in a variety of species [3]. Industrial nanoparticles, like those from iron mining, are particularly concerning for lung health, with small particles causing cell viability issues and increased apoptosis [4]. The toxicity of nanoparticles depends on both size and chemical composition. For instance, silver nanoparticles, used for their antibacterial properties, can cause cytotoxicity and disrupt mitochondria [5]. Carbon nanoparticles with ozone co-exposure can lead to inflammation [6], while cerium oxide nanoparticles, used in industrial catalysts, can induce apoptosis and oxidative stress [7]. Commonly used titanium dioxide in sunscreens and zinc oxide in sunscreens and electronics can cause lymphocyte alterations and dermal toxicity [8]. Zinc oxide nanoparticles can also cause lung inflammation and genotoxic responses [9,10,11,12]. The widespread presence of nanoparticles, including in human placenta, underscores the extensive and broad exposure to these materials in the environment [13]. Airborne nanoparticles can enter organisms through skin, eyes (cornea epithelium ocular [14]), ingestion (gut and intestine), and inhalation (nasal, oral, and pulmonary tissues). The respiratory system’s primary defense against NPs is physical barriers in epithelial cells, like those in alveoli, the respiratory tract, oral cavity, and nasal tissue. These barriers are characterized by tight junctions [15] and structures like villous mucosal structures to increase the biological interface area [16]. While many studies focus on respiratory uptake, nasal tissue is also crucial for filtering inhaled air, aided by various cell types including olfactory, respiratory, and lymphoepithelial cells [17,18]. Research on nasal tissue has explored its response to cancerogenic substances (nickel, wood, leather dust, ionizing radiation [19,20]), cigarette smoke’s pro-inflammatory effects [21], genotoxicity of e-cigarette propylene glycol [22], and Ni^2+^ ion cytotoxicity causing mitochondrial damage in nasal epithelial cells [23]. Recently, studies have shown the olfactory mucosa’s role in antiviral immunity [24].

The OECD guidelines provide in vivo and in vitro assays for assessing nanomaterial risks, including tests for lethality/toxicity, mutagenicity, chromosomal aberrations, and skin irritation. Alternative in vitro methods are increasingly used over animal tests, especially important for nasal uptake due to interspecies differences between rodents and humans [18,25]. In vitro tests focus on cell viability, genetic damage, proliferation, and barrier integrity, utilizing assays like dye exclusion [26,27], colorimetric [28,29], fluorometric [27,30], luminometric [31], and microscopic [32] as well as electrochemical methods [33]. Barrier models can provide data on barrier integrity and cytokine secretion [34], complementing established assays. Advances include lab-on-a-chip technologies for real-time metabolism monitoring [35], surface plasmon resonance sensors for protein measurement [36], and metabolic biosensors for lactate, glucose, and oxygen [37]. Our group has developed microfluidic platforms for nanotoxicological risk assessment, including models for vascular hyperpermeability [38], lung barriers [39], and placental trophoblast tissue [40]. Microfluidics allow more precise control and integration of electrochemical sensors for monitoring cell adhesion and barrier integrity, using techniques like trans-cellular electrical resistance (TER) measurements or electrical cell-impedance spectroscopy (ECIS) [41].

**Figure 1:**
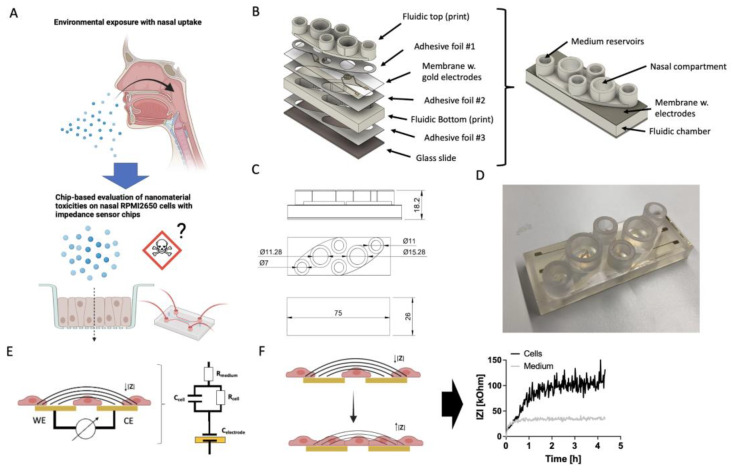
(**A**) Schematic representation of the proposed nasal barrier-on-a-chip platform idea based on RPMI2650 cells for the toxicological evaluation of potential cytotoxic effects of, i.e., zinc oxide nanoparticles, (**B**) exploded and assembled render views of the nasal barrier-on-a-chip monitoring modules with (**C**) technical drawings (magnitudes in mm) in top and side view, as well as (**D**) an example for a finished 3D-printed biochip with rapid-prototyped impedance disc electrodes located on porous cell-culture-treated membranes. The entire chip was manufactured with rapid prototyping procedures including SLA 3D printing for the top and bottom layers as well as structured double-sided biomedical ARcare adhesive layers structured by xurography. (**E**,**F**) Schematic of the bipolar cell substrate impedance spectroscopy method with a simplified circuit model that includes the capacitance of the electrode interface (C_electrode_), resistance and membrane capacitance of the epithelial cells (R_cell_ and C_cell_), as well as the resistance of the bulk electrolyte (R_medium_). (**F**) Schematic representation of cell adhesion dynamics captured by increase of impedance IZI over time when cells adhere and form cell–cell junctions.

Here, we demonstrate how rapid prototyping approaches can be applied to develop accessible and more affordable cartridge-based cell-based biosensing platforms for toxicological screenings. A combinatorial approach of 3D printing and xurography was used to establish a Transwell-like cartridge with integrated electrical impedance sensors. To replace cleanroom procedures for electrode fabrications, we developed a xurographic approach to produce gold thin-film disc electrodes on cell-culture-treated porous membranes using silicone shadow masking. Platform peripheries as well as the cartridge was manufactured by stereolithography (SLA) printing. To establish an integrated cell-based screening platform, the ECIS membranes were assembled into a system comprising two individual sensing units per slide using xurographically structured biomedical pressure-sensitive double-sided adhesives. The current platform development set out for a self-sufficient potential system to probe samples also in external scenarios. Consequently, the modular system comprised a temperature control unit, a physical humidity control, and a pneumatically controlled microinjection mechanism to simulate nano-environmental exposure and allow liquid handling, as well as solutions for miniaturized readout capabilities (i.e., mobile power source and a miniaturized potentiostat setup). After initial physicochemical as well as biological characterizations of zinc oxide nanoparticles of the European nanomaterial repository of the Joint Research Centre (JRC), we demonstrated how to use rapid prototyping approaches to evaluate acute nanoparticle exposure on human nasal epithelial barrier cells (RPMI2650; see Figure 1). Acute exposure of RPMI2650 cells with microinjections of 6 mM nanoparticle solution resulted in cytotoxicity, displayed by decreasing impedance slopes which were confirmed with qPCR and live–dead dye exclusion assays.

## 2. Materials and Methods

### 2.1. Prototyping of the Environmental Control Platform

The temperature control base plate comprised a plug-in power supply unit (Mean Well SGA60E24-P1J 24 V/DC 2500 mA DC2.1/5.5 mm; 1439220, Conrad, Wels, Austria), a separate control unit (dimensions: 171.9 mm × 120.9 mm × 55 mm, type IP66, 517-4235, RS Components, Gmünd, Austria) and the actual heating platform unit using Schneider Electric 70S2 Panel Mount Solid State Relay (60 V DC/5 A; 70S2-02-A-05-S 814-8197, RS Components), four mountable wire load resistors (2.7 Ohm ± 5%/25 W, 131-1255, RS Components) and a Wachendorff UR3274U5 PID controller (RS Components, 197814) equipped with a Pt100 thermo-sensor in ceramics housing (dimensions: 2 mm × 5 mm; 100-7528, RS Components). Specifications of the systems can be found in Table S1. Figure S1 shows the individual platform components including a 3D-printed cover with a bifurcated inlet for humidified air exposure (Figure S1A–D), the base plate with control unit (Figure S1E), as well as the circuit (Figure S1F) of the control and heating circuits.

The chip design was based on the format of a conventional object slide (dimensions: 26 mm × 76 mm), and adapted to the desired functionality to be able to sandwich membrane–electrode membranes in between two medium compartments to form a baso-lateral (lower) and apical (top) chamber compartment using Autodesk Fusion360. The chip components were printed using a Formlabs 3D printer (Model FORM3b), and Biomed Clear resin (Formlabs, Berlin, Germany). After printing, the top and bottom layer of the chip were bonded to an ipCELLCULTURE™ track etched Polyethylene terephthalate (PET) membrane (pore size: 0.4 μm, thickness: 12 μm, dimensions: A4 sheets; IT4IP) using biomedical double-sided pressure-sensitive adhesive tape (Adhesive Research, ARcare ^®^ 90106NB, Limerick, Ireland), and in the final assembly step top and bottom pieces were glued together. All individual parts of the chip were UV-C treated for 30–60 min for surface sterilization, and assembly was performed in sterile conditions inside a laminar flow hood (Herasafe KS-18, Thermo Fischer, Vienna, Austria).

### 2.2. Cell Culture, Nanotoxicology and Chip-Based Operations

Nasal RPMI2650 cells (Sigma Aldrich, 88031602, Vienna, Austria) originating from squamous cell carcinoma of nasal epithelium were cultured in Eagle’s Modified Essential Medium (EMEM, Sigma Aldrich, M0325) supplemented with 10% ELAREM™ FD (PL Bioscience, Aachen, Germany) and 1% Antibiotic Antimycotic solution (Sigma Aldrich, A5955, Vienna, Austria). The nasal epithelial cells were seeded at an optimized seeding density of 1 × 10^6^ cells/cm^2^, as previously described [42]. For experiments outside of a CO_2_ incubator, medium was supplemented with 100 mM HEPES buffer solution (1% *v*/*v* in complete culture medium).

For the evaluation of nanomaterial dose response, Presto Blue assay (Thermo Fisher, A13261) was performed according to the manufacturer’s instructions using a 10% mix to measure cell viability. Prior to the incubation of the nanoparticle-containing medium, RPMI2650 cells were allowed to adhere and form a barrier for at least 24 h in a 48-well plate. Nanoparticles were pre-incubated with 100% platelet lysate for 30–60 min to allow protein corona formation to reduce nanomaterial agglutination and then were adjusted to a final concentration of 6 mM in a complete culture medium. Readout was performed via fluorescence measurements (Excitation: 560 nm; Emission: 590 nm) using an EnSpire 2300 plate reader (PerkinElmer, Traiskirchen, AT).Characterizations of the heating performance of the stand-alone control platform for the prototyped biochip using two Omega CN7800 controllers equipped with PT-100 thermosensor covered with liquid Sylgard silicone elastomer sheathing were carried out to monitor the temperature change in the baso-lateral and apical compartments of the biochip. To characterize the cell barrier formation and integrity, potentiometric electric impedance spectroscopy was performed using a VMP3 potentiostat (BioLogic, Seyssinet-Pariset, France) with 100 mV peak-to-peak voltage up to a frequency of 10 Hz to 500 kHz with 10 points per decade. Biochips were connected with spring-loaded 3D-printed connectors to the potentiostat setup. Blank medium sensors were recorded as controls, and were double-checked after a cell-based experiment and enzymatic detachment of cells from the sensor to evaluate drifting of the rapid-prototyped membrane impedance sensors. Initial characterizations of RPMI2650 cell adhesion dynamics on the biochip prototypes mounted on the stand-alone control platform were conducted for a time course of 24 h against parallel acellular sensor controls. Prior to the nanotoxicological analysis, RPMI2650 cells were seeded at an initial seeding density of 1 × 10^6^ cells/cm^2^ and allowed to adhere and form a barrier for at least 24 h prior to nanoparticle exposure using the integrated prototyped micro dispenser unit, which was filled with 2 mL of ZnO nanoparticle solution at 6 mM concentration.

### 2.3. Design and Characterization of Xurographic Prototyped Gold Thin-Film Electrodes on Porous PET Membranes

**Figure 2:**
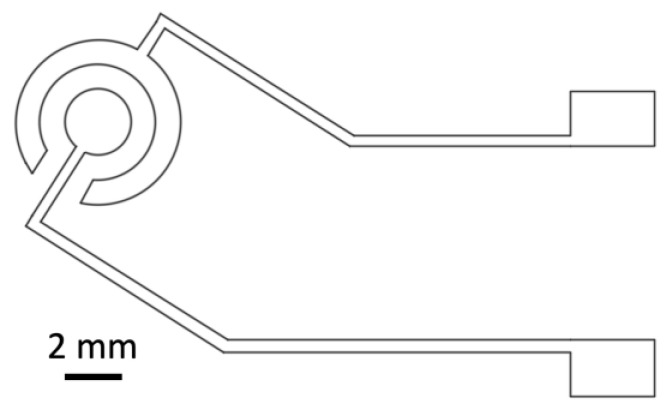
Technical drawing of gold thin-film disc electrode design used for the shadow mask xurography approach.

Xurographic fabrication of silicone sheets was conducted for clear silicone sheets (HT6240GK clear silicone sheets, MVQ Silicones, Weinheim, Germany), as described previously [43]. In brief, the shadow mask design for standard disc electrodes as shown in Figure 2 was cut into a 100 µm PDMS sheet using the Roland CAMM-1 GS-24 (Roland DB Benelux, Oevel, Belgium) cutter, set to a speed of 3 cm/second and 80 gf of force. The blade used was a Cemented Carbide Blade for fluorescent and reflective film and vinyl film (Roland ZEC-U1715).

**Figure 3:**
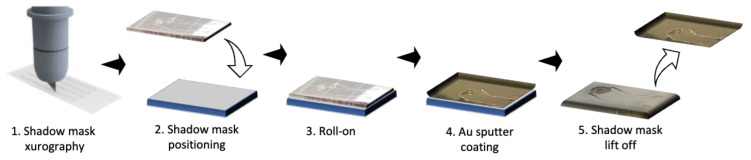
Xurographic rapid prototyping workflow for gold thin-film electrodes deposited on porous cell culture membrane structuring, roll-on, shadow masking, and lift-off of silicone-based structured shadow masks on PET membranes immobilized with 0.5% PVA on a glass carrier substrate.

Following the mask prototyping as shown in Figure 3, porous PET membranes were manually rolled onto a glass substrate spin-coated with polyvinyl alcohol as an adhesion layer. Next, a previously cut silicone sheet with the proposed electrode disc design was rolled onto the membrane after the removal of one PET liner. An 80 nm thin-film layer of gold was sputter-coated at a coating speed of 1.05 nm/s onto the layered assembly. After cooling down, the silicone shadow mask was removed by a manual lift-off procedure, and electrode membranes were stored under lab conditions until further use.

Basic characterizations of the thin-film disc electrodes were performed as quality control using a multimeter for short circuit detection, and an impedance spectrum recording was performed as a dilution series of phosphate buffered saline (0–10 mM) using a VMP3 potentiostat in potentiometric electric impedance mode with 100 mV peak-to-peak voltage up to a frequency of 10 Hz to 500 kHz with 10 points per decade. For evaluation of the miniaturized impedance readout capabilities, a CSX-64 multi-channel potentiostat module (Sciospec, Bennewitz, Germany) was also characterized.

### 2.4. Characterization of the Zinc Oxide Nanoparticle Reference Material

The Zinc oxide nanoparticles were acquired from the Joint Research Centre (JCR) nanomaterials repository (JRCNM62101a).

STEM Microscopy: ZnO particles were visualized employing a Magellan 400L XHR STEM operating at 20 kV in transmission mode STEM. Sample preparation involved drop-casting 10 μL of the sample onto a thin carbon-coated 200-mesh copper grid (Ted Pella, Inc., Redding, CA, USA), followed by air drying. STEM images of the as-received particles were utilized for size distribution measurements via Image J software (V1.54f).

Dynamic Light Scattering (DLS) and Zeta Potential: The size of NPs (nm) was determined using a Malvern Zetasizer Ultra instrument with a light source wavelength of 532 nm and a fixed scattering angle of 173°. Zeta Potential (mV) and conductivity (mS/cm) were measured using a Zetasizer Ultra (Malvern Instruments, London, UK). For analyses, 1 mL of the NPs solution (diluted 1:10 in Milli Q H_2_O) was placed in a cell, and measurements were carried out in a 1 cm optical path cell with precise temperature control (25 °C). The software was configured with parameters for refractive index, adsorption coefficient, and solvent viscosity at 25 °C. Each reported value represents the average of at least three independent measurements, all employing the Smoluchowski model [44,45].

### 2.5. Brightfield and (Immuno-)Fluorescence Imaging

For fluorescence imaging, cell samples were fixed with 4% paraformaldehyde in H_2_O and stored in PDMS with Ca^2+^ and Mg^2+^ ions at 4 °C. For immunofluorescence staining, the permeabilized cells (0.1% TritonX-100 in PBS; Sigma Aldrich, T8787, Vienna, Austria and Gibco, 10010023, Vienna, Austria) were labeled with a primary anti-ZO-1 monoclonal antibody (1:100 dilution; Abcam, ZO1-1A12, Vienna, Austria) and rabbit MUC1 monoclonal antibody (1:50; Abcam, EPR1023, Vienna, Austria); then the samples were blocked using animal-free blocking reagent solution (Animal-free Blocking Solution, Cell Signaling Technology, Liden, The Netherlands). For labeling, secondary Alexa Fluor 488—labelled recombinant goat anti-rabbit IgG (heavy Chain) and Alexa Fluor 555 goat anti-mouse IgG were incubated for 60 min prior to nucleic and phalloidin staining. All dyes and antibodies were diluted in PBS (Sigma-Aldrich) containing 0.5% animal-free blocking reagent (1:10 dilution) and were washed three times in between staining steps.

To determine viability, the Live/Dead Double Staining Kit from Sigma Aldrich (QIA76, Vienna, Austria) was used. An Olympus IX83 automated epifluorescence microscope was used for all microscopic imaging using a quad-bandpass filter.

### 2.6. mRNA Isolation and Gene Expression Analysis

In order to test the cells’ response to acute ZnO exposure at the transcription level, RT-qPCR analysis was conducted against the MUC1 and BAX genes. They were normalized against the expression of the housekeeping gene GAPDH (Primers see Table 1). mRNA was extracted from the samples using the innuPREP RNA Mini Kit 2.0 (IST Innuscreen GmbH, Berlin, Germany), following the manufacturer’s protocol, and then adjusted to an RNA concentration of 10 ng/mL. Next, the samples were retro-transcribed via the High-Capacity cDNA Reverse Transcription Kit (Applied Biosystems, 4368814, Vienna, Austria) and the Linegene FDQ-96A PCR machine by Bioer. Finally, qPCR was executed using the PowerTrack™ SYBR™ Green Master Mix (Applied Biosystems, #A46109), as intended by the manufacturer with the exception that a 1:5 diluted sample was utilized. The PCR was conducted with the QuantStudio™ 3 cycler (Applied Biosystems) and the QuantStudio™ Design&Analysis Software 1.3.1 (Applied Biosystems). All samples were stored at −20 °C until used.

### 2.7. Data Visulizations and Statistical Analysis

GraphPad Prism 10 was used for data visualization as well as statistical analysis of data. IC50 values were calculated using a fitted non-linear regression with variable slope (Hill; slope: −1). When comparing two groups, an unpaired t-test with Welch’s correction was used.

## 3. Results

### 3.1. Initial Characterization of the JCR Zinc Oxide Reference Material and the RPMI2650 Nasal Epithelial Cell Responses

In order to characterize the Zinc oxide nanoparticles reference material, we included basic physical–chemical characterization approaches including electron microscopy, dynamic light scattering, and zeta potential analysis (see Figure S2), and the biological effect of ZnO nanoparticles of two-dimensional cultures of RPMI2650 nasal epithelial cells using Presto blue metabolic viability assay. Transmission electron microscopy analysis revealed that the solid ZnO particles tend to aggregate in clusters of approximately 100–120 nm sized individual particles. More detailed DLS analysis revealed that 88.5% of the NPs in aqueous solution had a diameter of 140.9 ± 21.2 nm for individual NPs and bigger micron-sized aggregates of 1.5 µm. Zeta potential analysis of the ZnO particles revealed a positive zeta potential of 26.3 ± 1.36 mV in an aqueous solution at a pH value of 6.5. Analytical discrepancies between the reported 70–90 nm particle size of the reference material with our analyses may be attributed to particle aging, as well as stability and dissolution due to storage of the reference material.

**Figure 4:**
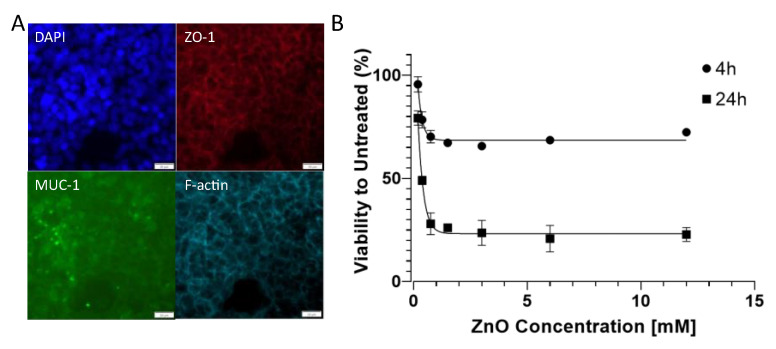
(**A**) (Immuno-)fluorescence micrographs of RPMI2650 cells stained with DAPI, anti-ZO-1/TRITC, anti-MUC-1/AlexaFluor 488 and AlexaFluor 647-labelled phalloidin. Bars represent 20 µm. (**B**) Viability analysis of RPMI2650 epithelial barrier cells exposed to increasing zinc oxide nanoparticle concentrations up to 12 mM in complete culture medium using Presto Blue assay for 4 h and 24 h post-exposure relative to the respective untreated controls.

Prior to the toxicological evaluation, we first investigated the presence of barrier-specific markers commonly associated with mucosa barriers. Immunofluorescence staining in Figure 4A confirmed the presence of mucus protein MUC-1, which is a characteristic protein involved in nasal mucosa barriers [46]. The tight junction protein ZO-1 localization, which was well co-localized with the phalloidin stain directed against f-actin, confirmed that at a seeding density of 1 × 10^6^ cells/cm^2^, the barriers showed continuous tight junction morphology, which points at an established confluent nasal cell barrier. This was particularly important as the proposed approach was aimed at rapid establishment of the cell-based nasal model with subsequent toxicological on-chip monitoring within a time window of 24 h. Figure 4B shows the nanotoxicological response of RPMI2650 cell barriers when exposed to cytotoxic 90 nm sized ZnO nanoparticles for a brief incubation period of 4 h compared to 24 h of nanomaterial exposure. For the 4 h incubation period, no IC50 value was detected; however, initial toxicity was observed for an IC10 value of 0.07 mM, where 10% of the RPMI2650 displayed cytotoxic events in the well plate-based PrestoBlue assay. After prolonged exposure for 24 h, an IC50 value of 0.32 mM was determined. This initial characterization of the nasal epithelial cell model revealed paracellular tight junction expression (ZO-1) that indicates a certain degree of barrier properties [15,16], while mucus protein-1 (MUC-1) was also expressed on this particular cell line, indicating a nasal mucosa character [46,47,48,49]. In case tighter barriers are needed for transport instead of toxicity studies, the maturation of RPMI2650 can be prolonged for several weeks to better resemble human nasal mucosa [50,51]. The response curve of RPMI2650 cells to various concentrations of ZnO in the current study concerning toxicity was in line with previous data of zinc oxide-based toxicity of nasal mucosa Transwell-type assays around 0.5 mM for 24 h post-exposure [42].

### 3.2. Rapid Prototyping of Thin-Film Gold Impedance Disc Electrodes on Porous Cell-Culture-Treated PET Membranes and Sensor Response Behavior

**Figure 5:**
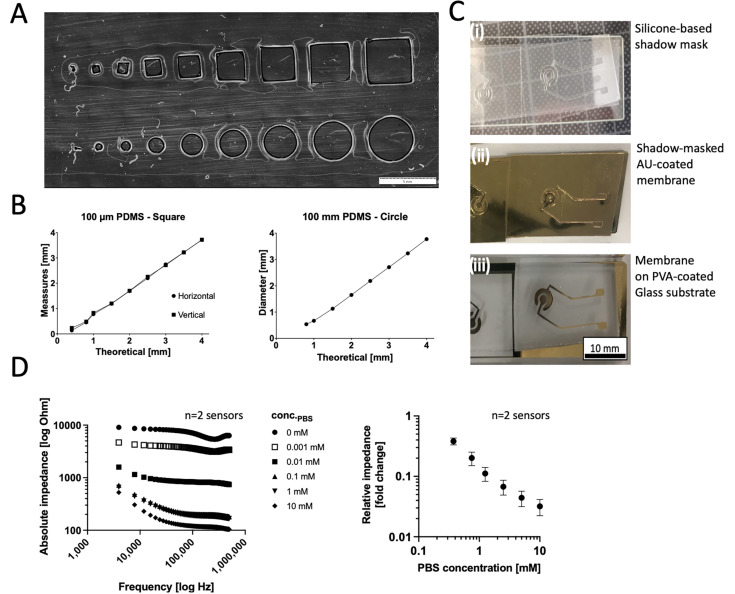
(**A**,**B**) Initial characterizations of the resolution limit of square and circular structures on 250 µm thick silicone sheets structured on a PET lining layer. (**C**) Images of (i) silicone shadow masks, (ii) shadows masks on a porous Pet membrane after AU sputter coating, and (iii) the disc electrode structures after mask lift-off on porous Pet membrane substrates immobilized on a PVA-coated glass carrier material. (**D**) Effect of ionic strength of PBS (serial dilution range 10–0 mM) on the frequency behavior and relative response of the rapid-prototyped disc electrodes (*n* = 2).

To test the precision of the xurographic shadow masks, test structures were analyzed, as shown in Figure 5A. Figure 5B depicts the relationship between the design’s theoretical magnitudes and the measured values and shows high agreement in designs with structure sizes above 1 mm. Figure 5C shows the individual steps of the xurographic electrode fabrication with initial characterizations of the Bode plots of Figure 5D revealing a normal behavior of thin-film electrodes directly proportional to the ionic strength of serial PBS dilutions at higher high-frequency region (10 kHz–500 kHz). The impedance spectra exhibit a pure resistance characteristic, which is mainly related to the difference in ion concentration in PBS solution with a decreasing impedance value with an increasing saline concentration from 8.10 ± 1.57 Ohm to 0.25 ± 0.027 kOhm for 10 mM and 0 mM buffer, respectively.

**Figure 6:**
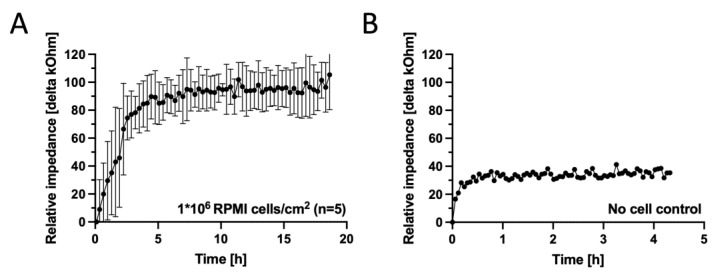
(**A**) Impedance–time curves of Au thin-film disc electrodes on porous cell-culture-treated PET membranes fabricated for a seeding density of 1 × 10^6^ RPMI2650 nasal epithelial cells/cm^2^ (*n* = 5 disc electrodes, grey and black signals represent the raw signal and the rolling average, respectively) in comparison to (**B**) a sensor response of a disc electrode incubated complete culture medium for the next 180 h (*n* = 1 electrode) at 37 ± 1 °C platform temperature. Data were baseline subtracted against medium blank impedance after initial equilibration of 30 min.

In order to test the biological performance of the fabricated membrane electrodes, next impedance spectroscopy measurements were performed over time for ECIS sensors in the presence and absence of the RPMI nasal cell model. Figure 6A depicts the impedance–time traces of the cell layer formation over the electrodes. An initial steep increase in impedance over the first 5 h to 86 ± 12 kOhm at a slope of 0.28 kOhm min^−1^ is attributed to the conventional adhesion dynamic of the RPMI2650 cells at a seeding density of 1 × 10^6^ cells/sensor. A plateau phase for another 15 h, peaking at 95 ± 3.7 kOhm with an incline of 0.01 kOhm min^−1^, indicated good and confluent coverage of the entire sensor surface and barrier maturation. Figure 6B shows a membrane disc electrode control incubated with cell culture media in the absence of RPMI cells, plateauing at a relative impedance of 35 kOhm at an incline speed of 0.13 kOhm min^−1^, confirming that the rapid-prototyped disc electrodes are sensitive to cell adhesion and coverage of RPMI2650 epithelial cells. Cell adhesion monitoring as demonstrated here for membrane-deposited ECIS electrodes is a well established cell analysis method [52] ideally suited for time-resolved rapid identification of acute and chronic cell toxicity. PDMS xurography allowed fast production of meso-scale thin-film disc electrode structures on porous cell culture membranes for rapid impedimetric slope readouts of epithelial cells as a matter of cell adhesion altercations to indicate cell stress and cytolytic responses [38,39,52,53]. For electrode types requiring higher resolutions, we previously also demonstrated a photolithographic method for smaller structures at the cost of reduced user accessibility, higher infrastructural costs, as well as higher time requirements [41].

### 3.3. Characterization of the Temperature and Humidity Control Capabilities of a Stand-Alone Biosensing Platform

As the current project aimed to develop an analytical system with rapid prototyping means to evaluate nanotoxicology of epithelial models outside of conventional cell culture infrastructures, we next investigated our platform’s capabilities to control the environment of the prototyped biochips, including temperature and humidity control capabilities (see Figure S3A for design details).

Figure S3B–D shows that the resistive heating under the biochip reached a set-point of 88 °C within 14.9 min at a 4 °C/min rate, achieving 37.5 °C in 9.5 min and stabilizing at 37 ± 0.5 °C for several hours. At a physiological set-point of 36 °C, apical and basal compartments warmed to 36 °C in 40 min with a 60 min preheating protocol ensuring uniform temperatures before cell seeding. Notably, a temperature of 36 °C corresponds to a physiological temperature found in external nasal tissue [47]. Figure S3E shows that an IH18 nebulizer module can maintain high humidity with a 0.36 ± 0.01 mL/min evaporation rate, while pressure tests showed increased nebulization rates of 0.43, 0.46, and 0.47 mL/min at 1, 1.25, and 1.5 bar, supporting environmental control for mucosal barrier system testing. Figure S4 shows the basic design of the prototyped medium dispensing module that was developed to simulate environmental exposure to nanoparticle suspensions at a minimal exposure rate of 20 µL s^−1^. To demonstrate that the ECIS membrane setup with heating and peripheries can also be used as a stand alone platform, Figure S5A shows the basic setup, comprising a Chuwi Windows 11 control mini-PC in combination with a CSX-64 multichannel potentiostat unit powered by a 1200 W/120 V uninterruptible power supply unit. Figure S5B,C confirms the normal behavior of thin-film electrodes, again directly proportional to the ionic strength of serial PBS dilutions in high-frequency regions, exhibiting a pure resistance characteristic similar to the benchtop VMP3 potentiostat. Notably, the relative variability of the mobile potentiostat was 10-fold higher compared to the benchtop system, which indicates some drawbacks of the miniaturized platform regarding precision.

### 3.4. Rapid Analysis of Acute Toxicity on the Rapid-Prototyped Monitoring Platform for RPMI2650 Cells Exposed to Cytotoxic Levels of Zinc Oxide Reference Nanoparticles

**Figure 7:**
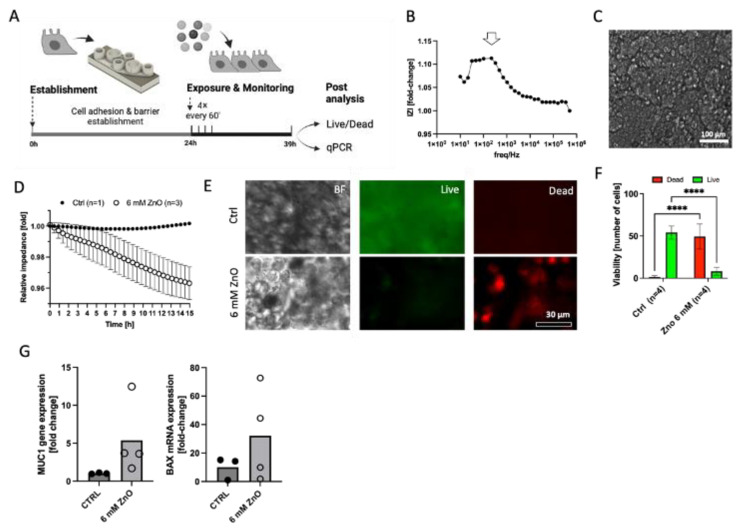
(**A**) Schematic representation of the final concept study for nanoparticle toxicity of JRC zinc oxide reference particles on RPMI2650 nasal epithelial cells monitored on the finished demonstrator assembly of the environmental monitoring platform. (**B**) Sensitivity of the thin-film gold disc electrodes on cell-culture-treated porous PET membranes expressed as delta fold change for electrodes prior to and after overnight cultivation with (**C**) a dense RPMI2650 cell layer, as shown in the representative brightfield image. (**D**) Normalized impedance–time curves of RPMI2650 cells 24 h post-seeding when exposed to 6 mM ZnO nanoparticle solution (*n* = 3) in comparison to control medium (*n* = 1) for an observation period of 15 h post-exposure. (**E**) Representative live/dead fluorescent micrographs and (**F**) quantitative viability analysis of exposed (*n* = 4 biochips) and non-exposed (*n* = 4 biochips) RPMI2650 nasal epithelial cells after 15 h of exposure on the biosensing platform. Data are expressed as mean ± SD. (Two-way ANOVA, **** *p* < 0.001). (**G**) Respective RT-qPCR analysis of non-exposed and exposed RPMI2650 cells after nanomaterial exposure on the monitoring platform for target genes MUC1 and BAX. Data are expressed as fold change relative to the GAPDH housekeeping gene.

As a final proof of concept, we exposed the on-chip RPMI2650 epithelial model to ZnO nanoparticles at a cytotoxic concentration of 6 mM, while inside the biochip platform, by 4 hourly dispensing protocols and evaluated the acute cytolytic activity with the prototyped sensing cartridges. As seen in Figure 7A, the cells were seeded (1 × 10^6^ cells/cm^2^) and cultured for 24 h inside the generated artificial environment in order for them to attach and form a barrier. Next, the cells were exposed to 50 µL of ZnO solution every hour for 4 h for a total concentration of 6 mM. Then, the cells were incubated for another 12 h before analysis. After an initial 24 h cultivation, the sensitivity of the gold electrodes was tested by recording the impedance over a wide frequency range. Figure 7B shows the frequency of 0.8 kHz to be optimal for measurements of cell impedance. In Figure 7C, it is possible to see the RPMI2650 cells after 24 h of cultivation inside the biochip, supported by the artificial environment. This brightfield image shows how dense the cells grow in order to form a tight barrier. Cell impedance response to ZnO exposure can be seen in Figure 7D. While the impedance slope for an untreated nasal biosensor sample stayed stable over the 15 h period with 1 ± 0.0016-fold impedance signal change, the impedance curves of the treated epithelial samples decreased with a declining slope of 2.9 ± 3.8 Ohm/hour. Figure 7E confirms the amount of live (green fluorescence) and dead (red fluorescence) cells after 15 h of ZnO exposure. The additional quantitative image analysis in Figure 7F reveals a high RPMI cell viability of 97 ± 2.9% for untreated nasal epithelial cells exposed to plain culture medium injections, while a decline of cell viability down to 14.6 ± 8.9% (two-way ANOVA, *p* < 0.0001) confirmed significant cell necrosis by acute ZnO exposure within a 15 h analysis window. Figure 7G indicated that both apoptotic markers MUC1 and BAX displayed a tendency to be more expressed in NP-treated RPMI2650 cells. By measuring cell surface impedance directly on the porous membrane instead of TER, we could rapidly correlate the declining slope of the impedance curve directly proportional epithelial cell cytolysis, which, in turn, displayed mucosa cell-specific stress markers (MUC1) and upregulation of the apoptosis marker BAX [16,46,54]. Notably, such a straight-forward biosensor platform technology as demonstrated here can not only be used for toxicity screens (i.e., acute exposure scenarios), but potentially also to investigate the regenerative capacity of mucosal cells inside lab-on-a-chip systems, as demonstrated by our group for lung biochip models previously [39].

Our rapid prototyping method has shown that standard disc electrodes on cell-culture-treated porous PET membranes enable quick cell adhesion monitoring within 15 h for non-toxicological studies. Using pressure-sensitive biomedical adhesive tapes, we easily joined the electrode membrane to a bottom compartment holding 3 mL of culture medium and a top 500 µL compartment, simulating a Transwell’s basic functionality using our xurographic bonding technique which proved useful for 3D-printed components. It facilitates the printing and assembly of components in a laminar flow hood, completing prototype fabrication within 24 h, from 3D printing initiation to assembly. This approach offers a cost-effective, rapid method to study nasal mucosa adhesion and cytolytic events, among other potentially interesting epithelial cell models (i.e., oral, bronchial, dermal, alveolar, or ocular barriers), for preliminary screenings of acute toxicities.

## 4. Conclusions

Over the last twenty years, advancements in biochip and microsystems engineering have led to the development of increasingly complex cell-based microfluidic systems. These systems blend micro total analysis systems, lab-on-a-chip, organ-on-a-chip technologies, and modern tissue engineering, with organ-on-a-chip models being particularly effective in creating three-dimensional human tissue models. While these models are advancing rapidly in biological accuracy, technological development, particularly in automation and monitoring, is lagging due to limitations in liquid handling and the instability of long-term enzymatic metabolic sensors. To address the ‘chip-in-a-lab’ versus ‘lab-in-a-chip’ debate, newer microfluidic approaches incorporate more stable optical or electrochemical sensors. However, these still require extensive lab equipment like incubators and control stations for microfluidic pumps. For environmental exposure scenarios, like those in our current study, small-scale, portable platforms are being developed to facilitate fieldwork, shifting from traditional methods of capturing airborne toxicants. Our current study aims to establish a nanotoxicological screening system using rapid prototyping technologies, making mucosal barrier chips equipped with biosensors accessible and affordable for a wider scientific community. Notably, the infrastructural requirements needed for most microfabrication require tedious cleanroom and expensive techniques, which allow for the precise replication of devices via photolithographic patterning and soft lithographic molding [48]. We envision testing our platform for acute toxicity monitoring in the field as a rapid qualitative approach prior to more thorough laboratory techniques such as mass spectrometry, ELISA, qPCR, and others. The current limitations of the system are mainly based on the utilization of a benchtop potentiostat, which we demonstrated here to be easily replaceable by smaller solutions for impedance readout [55].

Overall, affordable rapid prototyping methods including 3D printing, xurography, and silicone-based shadow masking are ideally suited to create versatile and affordable platform technologies aimed towards acute cytotoxic risk assessment studies [53,55,56,57]. We demonstrated in a proof of concept how the proposed prototyping pipelines are feasible to create human cell models for cytotoxic evaluation approaches in environmental (nano-) toxicology, where the ultimate goal may be not expensive and quantitative assaying but simple and affordable pre-screening tools for environmental risk assessment, especially when coupled in the future with unmanned vehicle applications. A major task ahead from a technological perspective will also concern (i) integration of more sensing units on a single device, (ii) air manipulation and handling to control sampling of air-borne toxicants within the apical compartment, (iii) wireless data transfer and/or local storage, as well as (iv) a lighter mobile energy management to provide the proposed platform the mobility needed to perform toxicological analyses not only in the lab but also field environments.

## Figures and Tables

**Table 1:** Human primer sequences.

Target	Species	Forward Sequence	Reverse Sequence
GAPDH	Human	GGAGTCCACTGGCGTCTTCAC	GAGGCATTGCTGATGATCTTGAGG
MUC1	Human	GGTCATGCAAGCTCTACCCC	AGCTGGGCACTGAACTTCTC
BAX	Human	TTCATCCAGGATCGAGCAGG	GGAAAAAGACCTCTCGGGGG

## Data Availability

Data will be provided by reasonable email request by the corresponding author.

## Supplementary Materials

The following supporting information can be downloaded at: https://www.mdpi.com/article/10.3390/bios14020107/s1, Figure S1: Technical drawings of (A–C) the platform cover, and (D) the bifurcated top inlet for humification. (E) Images of the external control unit and the heating base plate of the platform with (F) circuit drawings of the resistive heating approach; Table S1: Environmental control platform specifications; Figure S2: Schematic drawing of the shadow mask structures for the xurographic prototyping of silicone shadow masks for gold sputter coating; Figure S3: Schematic drawings of the (A–C) top level accessories with (D) connector to the external nebulizer humidification unit; Figure S4: (A,B) Schematic drawing of the computer-controlled Elveflow Flow-EZ setup for pneumatically actuated dispensing unit using a rapid-prototyped medium flow chamber.
