# Supplementary material for: Using Rapid Prototyping to Develop a Cell-Based Platform with Electrical Impedance Sensor Membranes for In Vitro RPMI2650 Nasal Nanotoxicology Monitoring
Source: Biosensors (Basel). 2024 Feb 18;14(2):107. doi: 10.3390/bios14020107 (PMC10886737; doi:10.3390/bios14020107)
Supplement: Supplementary file 1 [file biosensors-14-00107-s001.zip › biosensors-2807240-supplementary.pdf]

biosensors

MDPI

Article

Supplementary Material File - Using Rapid Prototyping to Develop a Cell-Based Platform with Electrical Impedance Sensor Membranes for In Vitro RPMI2650 Nasal Nanotoxicology Monitoring

Mateo Gabriel Vasconez Martinez [1,†], Eva I. Reihs [1,2,†], Helene M. Stuetz [1], Astrid Hafner [1], Konstanze Brandauer [1], Florian Selinger [1], Patrick Schuller [1], Neus Bastus [3], Victor Puntes [3], Johannes Frank [4], Wolfgang Tomischko [4], Martin Frauenlob [1], Peter Ertl [1,4], Christian Resch [5], Gerald Bauer [5,6], Guenter Povoden [6,7,8,9] and Mario Rothbauer [1,2,*,†]

[1] Institute of Applied Synthetic Chemistry, Faculty of Technical Chemistry, Technische Universitaet Wien, Getreidemarkt 9/163, 1060 Vienna, Austria; mateo.martinez@tuwien.ac.at (M.G.V.M.); eva.reihs@tuwien.ac.at (E.I.R.); konstanze.brandauer@tuwien.ac.at (K.B.); patrick.schuller@tuwien.ac.at (P.S.); martin.frauenlob@tuwien.ac.at (M.F.); peter.ertl@tuwien.ac.at (P.E.)
[2] Karl Chiari Lab for Orthopaedic Biology, Department of Orthopedics and Trauma Surgery, Medical University of Vienna, Währinger Gürtel 18-22, 1090 Vienna, Austria
[3] Catalan Institute of Nanotechnology, UAB Campus, 08193 Bellaterra (Barcelona), Spain; neus.bastus@icn2.cat (N.B.); victor.puntes@icn2.cat (V.P.)
[4] Institute of Chemical Technologies and Analytics, Faculty of Technical Chemistry, Vienna University of Technology, Getreidemarkt 9/164, 1060 Vienna, Austria; johannes.frank@tuwien.ac.at (J.F.); wolfgang.tomischko@tuwien.ac.at (W.T.)
[5] Science, Research, and Development Division; Austrian Federal Ministry of Defence; 1090 Vienna, Austria
[6] CBRN-Defence-Centre, Austrian Armed Forces, 2100 Korneuburg, Austria
[7] Institute of Inorganic Chemistry, University of Technology, Stremayrgasse 9/IV, 8010 Graz, Austria
[8] Department for Legal Philosophy, Law of Religion and Culture, University Vienna, Freyung 6, 1010 Vienna, Austria
[9] Institute of Environmental Biotechnology ,University of Natural Resources and Life Sciences (BOKU), IFA Building 1, Konrad-Lorenz-Straße 20, 3430 Tulln an der Donau, Austria
* Correspondence: mario.rothbauer@tuwien.ac.at or mario.rothbauer@meduniwien.ac.at
† These authors contributed equally to this work.

1. Content. Figure S1; Figure S2; Figure S3; Figure S4; Figure S5; Table S1

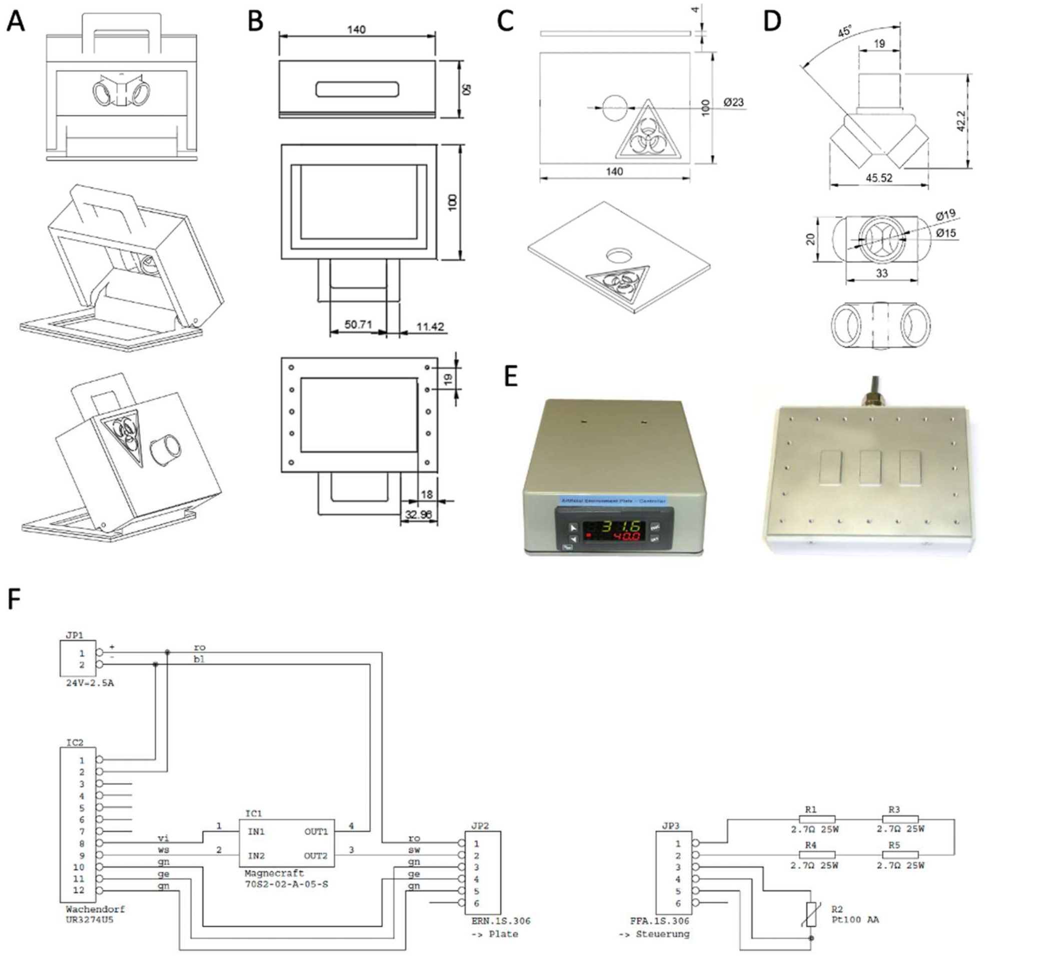

Figure S1. A) Technical drawings of A-C) the platform cover, and D) the bifurcated top inlet for humification. E) Images of the external control unit and the heating base plate of the platform with F) circuit drawings of the resistive heating approach.

Table S1. Environmental control platform specifications.

Maximum	
Voltage/current	24 V/ max. 2.5A
Heating capacity	50W
Heating velocity	4.4°C/min
Heating temperature	90°C
Stability	±0.03°C (t=3 h)

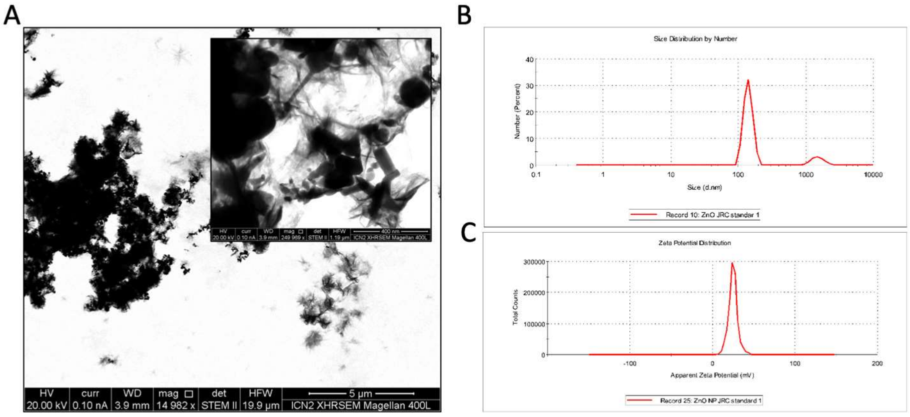

Figure S2. Characterization of the zinc oxide reference nanoparticles NM62101a of the JRC nanomaterial repository including **A)** transmission electron microscopy, **B)** dynamic light scattering (DLS) analysis and **C)** zeta potential analysis dispersed at a concentration of 8mg/mL in Milli-Q water.

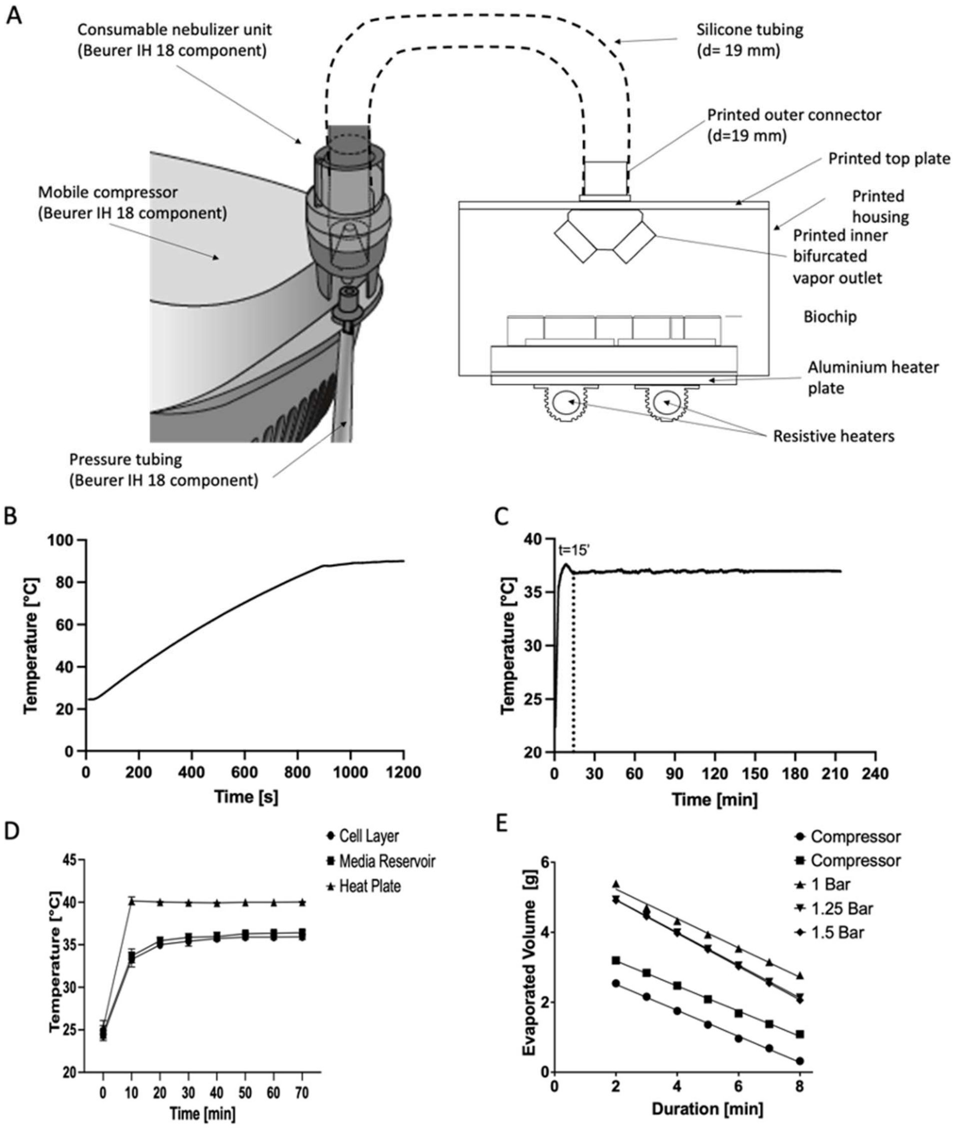

Figure S3. A) Overall concept of the physical humidification of the top part and the temperature control of the bottom part of the environmental control platform with **B)** maximal heating speed, **C)** transient behavior of the resistive heater setup, and, **D)** offset temperatures between the bottom

heat plate and the medium filled baso-lateral medium reservoir and apical side of the electrode membrane for cell adhesion. **E)** Evaporation volume analysis of the chamber humidification strategy by physical nebulizing between the modular commercially available compressor unit of the Beurer IH18 setup compared to an in-house wall-mounted pressure line.

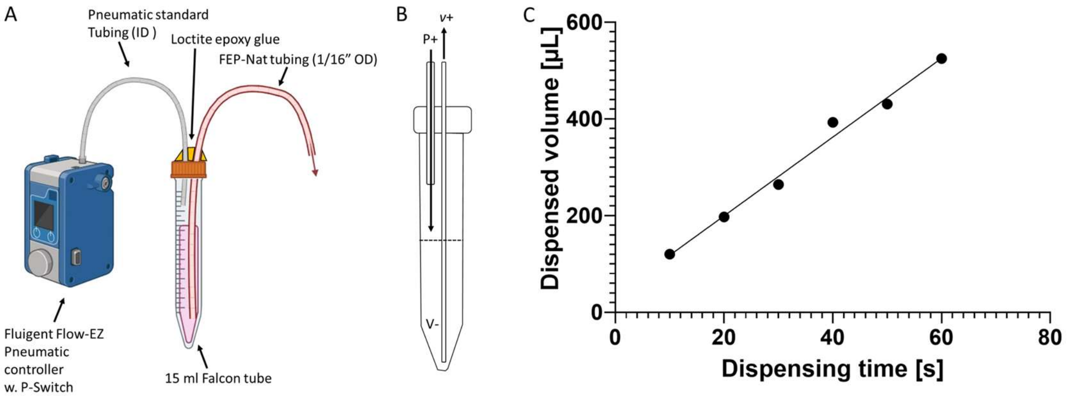

Figure S4. (A) Schematic drawing and cross-section view (B) of the micro-dispenser and (C) the characterization of the dispensed volume over time.

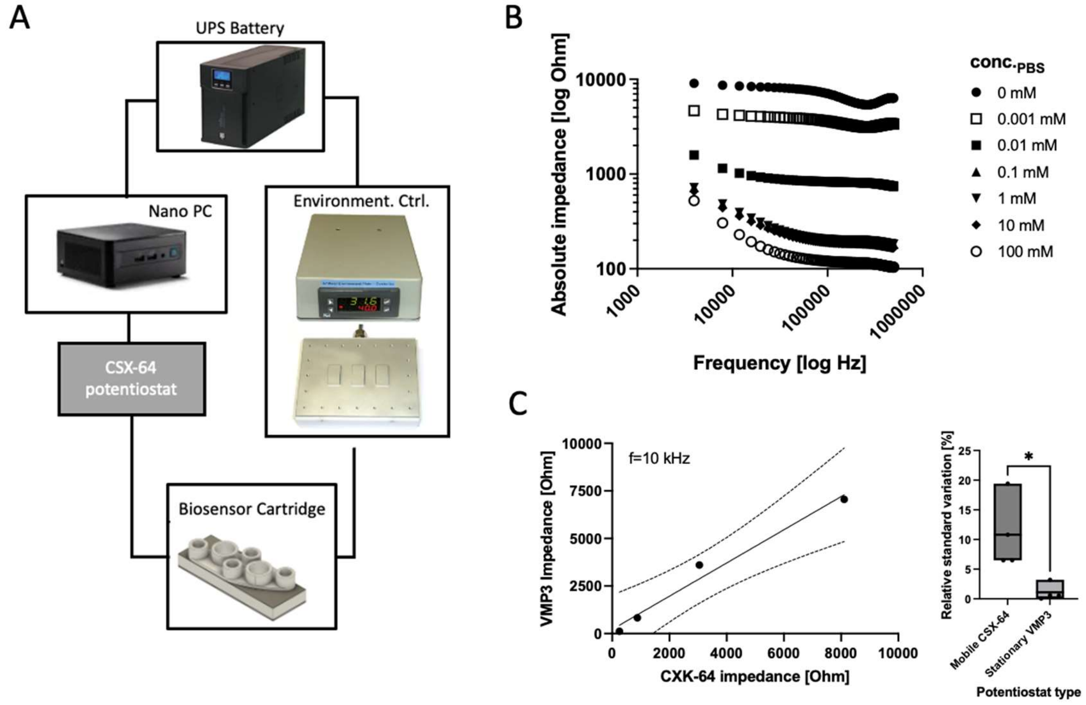

Figure S5. (A) Schematic drawing of the mobile stand-alone biosensing setup comprising an uninterruptible power supply (USP), a nano-PC, a mobile CSX-64 multi-channel impedance module, the environmental control base platform, and the prototyped cell-based biosensor platform. (B, C) Frequency behaviour and comparative sensor response of the VMP3 potentiostat and the CSX-64 potentiostat module for increasing PBS concentrations between 0 and 10 mM with analysis of the relative standard deviation of the respective impedance read-outs (unpaired t-test, n=4 readings).
